# The Geroscience Perspective on One Health

**DOI:** 10.1093/biosci/biaf080

**Published:** 2025-06-26

**Authors:** Colin Farrelly

**Affiliations:** Department of Political Studies, Queen's University in Kingston, Ontario, Canada

**Keywords:** One Health, aging, exposome, geroscience, environmental health

## Abstract

The One Health concept has been invoked to foster collaborations at the intersection of human, animal, and environmental health to address zoonotic disease risk. But the One Health concept is also helpful in framing and addressing the public health imperative to prevent and delay the onset of the chronic diseases of late life, to extend healthy life expectancy in ways that are sustainable and beneficial to animal and environmental health. This is an urgent global health imperative because healthy life expectancy is not increasing at the same pace as life expectancy, leading to more years being lived in poor health. This Forum article advances the Geroscience Perspective on One Health, a framework that draws attention to the interdependence of healthy human aging and animal and environmental health by emphasizing the importance of pursuing nonpharmacological (environmental and lifestyle) and potential pharmacological (gerotherapeutics) interventions to increase healthspan.

The One Health concept has been invoked to foster collaborations at the intersection of human, animal, and environmental health, and to date, the greatest acceptance of the concept has been in the developing world, where there are significant health impacts from the control of infectious diseases (Atlas 2013). Human and veterinary medicine, for example, are directly linked by their mutual interests in combatting zoonotic diseases, such as tick-borne diseases (Dantas-Torres et al. 2012). And the World Health Organization's (WHO) *One Health High-Level Expert Panel Annual Report*  *2023* emphasizes the importance of preventing zoonotic spillover (WHO 2024). But the One Health concept is also helpful in framing and addressing the public health imperative to prevent and delay the onset of the *chronic diseases* (e.g., heart disease, cancer and Alzheimer's) most prevalent in later life, to extend the healthy life expectancy of the global population in ways that are sustainable and beneficial to animal and environmental health. This is an urgent global health imperative as healthy life expectancy is not increasing at the same pace as life expectancy, leading to more years being lived in poor health (GBD 2019 Demographics Collaborators 2019, Lee et al. 2024). Over the past 20 years, in 183 WHO member states, the healthspan–lifespan gap has widened to 9.6 years (Garmany and Terzic 2024).

This Forum article begins by addressing the themes of imagination and idealism in medical science, a topic that was the focus of concern in Christian Herter's 1910  *JAMA* Address entitled “Imagination and Idealism in the Medical Sciences” (Herter 1910, Farrelly 2023a). Herter made a distinction between humanitarian medicine and experimental science and emphasized the plasticity of science (Farrelly 2023b). He maintained that science is “constantly subject to revision of its facts and ever ready to welcome new interpretations of old facts, as well as new discoveries, both great and small.” In this Forum, Herter's insights on imagination and idealism are applied to the One Health concept, to illuminate how One Health can reshape our perception of, and our ability to redress, the health risks of both infectious and chronic disease in ways that take seriously the interconnections between human, animal, and environmental health. The Forum then surveys some of the complementary empirical insights at the intersection of environmental science and gerontology, which reveal a distinctive imaginative perspective on One Health, which I call the *Geroscience*  *Perspective on One Health* (hereafter GPoOH) (figure 1). GPoOH draws attention to the interdependence of healthy human aging and animal and environmental health.

**Figure 1.**
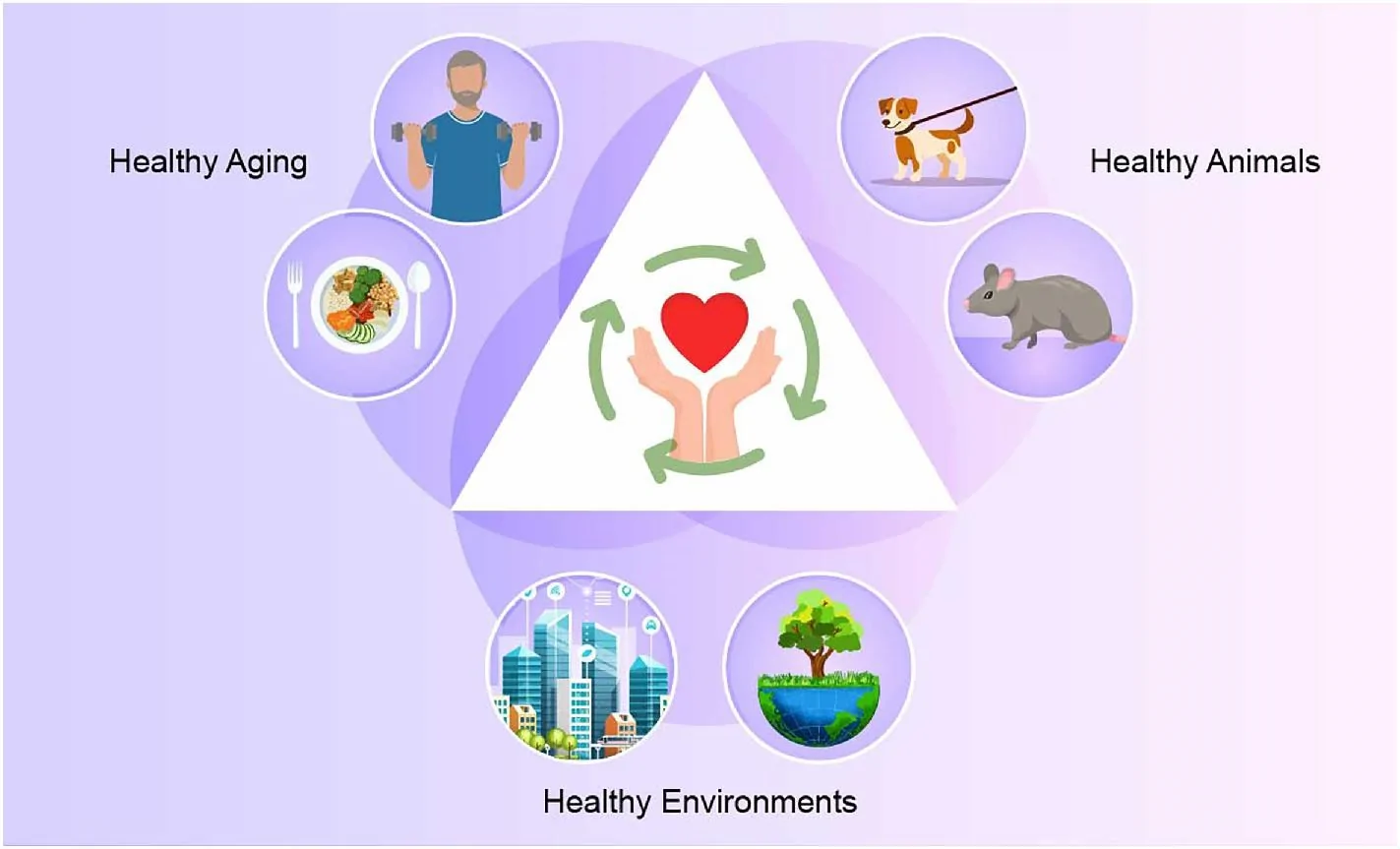
The Geroscience Perspective on One Health.

GPoOH can be helpful in communicating the moral imperative to slow biological aging via a diverse range of safe and effective interventions, including not only nonpharmacological interventions such as environmental and lifestyle modifications but also potential pharmacological interventions (*gerotherapeutics*) that may increase the human healthspan (figure 2). Healthspan refers to the period of life spent in good health, free from the chronic diseases and disabilities of aging (Kaeberlein 2018). The drug innovation dimension of biomedical gerontological innovation can create some communicative challenges for the GPoOH, given the prevalence of climate change ageism (Ayalon and Roy 2023). GPoOH can help inspire narratives of intergenerational solidary, rather than conflict, by directing our attention and collective energies toward effective and creative solutions to improve both human and planetary health. The latter encompasses concern for the former but is more expansive because it is defined as the health of human civilization and the state of the natural systems on which it depends (Whitmee et al. 2015).

**Figure 2.**
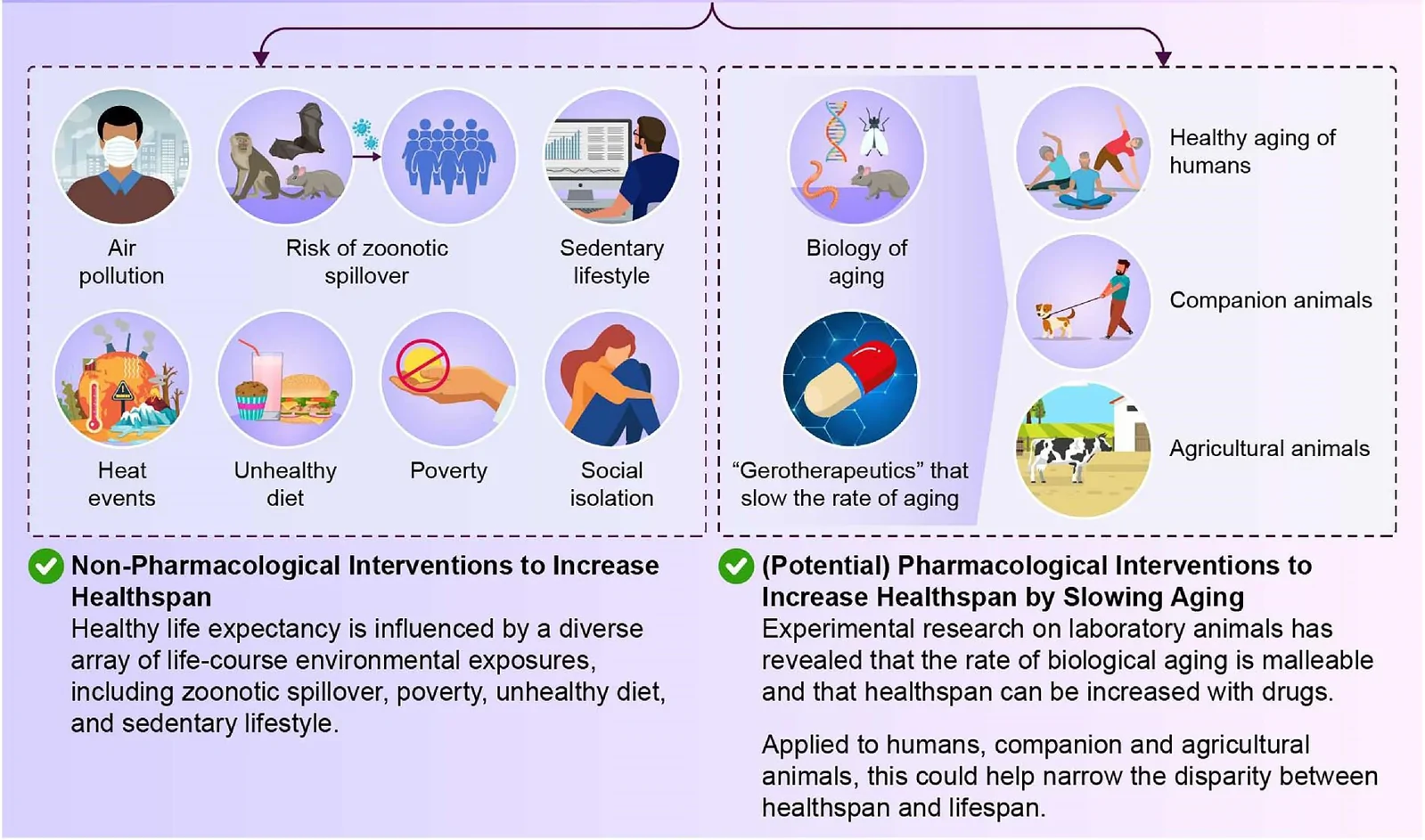
The foundational premise of the Geroscience Perspective on One Health: Healthy human aging is interdependent with animal and environmental health.

## Idealism and imagination in medical science

In his 1910 *JAMA* Address (Herter 1910), the pathologist Christian Herter made an important distinction between two ways imagination and idealism can be invoked in the medical sciences, and both can be transposed to the One Health concept. These two approaches are humanitarian medicine, which emphasizes the obvious and direct paths of ameliorating human suffering, and curiosity-oriented medicine, which explores pure science (e.g., chemistry) and the experimental laboratory to reveal the indirect ways of winning the citadel of health promotion.

The history of the One Health concept is long and varied. Evans and Leighton (2014) contend that its origins and development can be traced back to Aristotle through to zoobiquity (Natterson Horowitz and Bowers 2012) and that the interdependence of humans, animals, and respect for land and water has been an intrinsic part of the culture and spiritual beliefs of many ancient civilizations and Indigenous people. The One Health concept's focus on mitigating the risks from zoonic spillover is arguably the paradigmatic example of humanitarian medicine's collaborative approach to One Health. Humanitarian medicine's focus on the direct and obvious paths to ameliorating the human suffering and mortality from infectious disease reveals how critical the complex interconnections between human, animal, and environmental health are. Human populations living in close proximity to animals and their environments, where infectious disease risks may be prevalent, are themselves at risk of poor health outcomes because diseases can pass from animals to humans (and vice versa). These infectious diseases range from rabies and West Nile virus to anthrax, COVID-19, Ebola, and Lyme disease. Protecting all animals, domestic and wild, from disease and environmental hazards can therefore help protect the health of human populations.

As important as humanitarian medicine is, Herter argued that it can be a constraint on imagination and idealism in medical science. He maintained that advocates of humanitarian medicine are often resistant to adopting a path that entails taking a less obvious route to health promotion. Instead, they prefer to remain steadfast in their commitment to only pursuing the already tried and tested ways of ameliorating human suffering. However, this perpetuates a rigid, fixed mindset toward medical progress. It leaves little room for medical innovation. Herter complained that medical textbooks and lectures were “conservative and dispose us strongly to the notion of fixity of facts, making our minds statical in conception.”

Herter himself was an experimentalist and had a laboratory built into his home (New York Times 1910), and he cofounded the *Journal of Biological Chemistry* in 1905. In his 1904 book *The Influence of Pasteur on Medical Science* (Herter 1904), based on his address delivered to the medical school of Johns Hopkins University, Herter emphasized how Louis Pasteur's training in chemistry and physics enabled Pasteur to observe things and processes minutely and to interpret those observations (Herter 1904), as well as to have the confidence to apply these sciences to the solution of biological and medical problems.

Pasteur's doctoral thesis was on the crystals created in wine fermentation (Berche 2012), a topic that had no obvious direct medical application. But eventually, Pasteur's research led to pasteurization, and he also developed vaccines for rabies and anthrax. Pasteur's story is an exemplar illustration of how experimental science, which integrates insights from diverse fields of study, can enable the health sciences to redress the health vulnerabilities that arise from the interconnectedness of human, animal, and environmental health. Herter argued that the slow, gradual exploration of pure science, through the experimental method, is how major innovations in medical science are made.

Experimental science, in contrast to humanitarian medicine, embraces a vision of science as *plastic*, by which Herter meant it was subject to revision of its facts and ready to welcome new interpretations of old facts, as well as new discoveries. The One Health concept embodies this plastic view of science, becaue it enables new empirical discoveries—ranging from climate science to the biology of aging—and medical innovations to reshape the concerns, as well as policy prescriptions, of the One Health concept. In particular, it has the plasticity needed to expand its concern for abating zoonotic spillover to the broader goal of promoting healthy aging in ways that can be environmentally sustainable, and even beneficial to planetary health. This is GPoOH, which has both a humanitarian and a more experimental expression. The former refers to the nonpharmacological interventions of preventative medicine, whereas the experimental expression of GPoOH emphasizes the imagination and idealism of developing gerotherapeutic drugs which target the biological pathways involved in aging with the aim of reducing the burden of aging-related diseases and increasing lifespan and healthspan (Kulkarni et al. 2020, Le Couteur and Barzilai 2022, Guarente et al. 2024, and Mandelblatt et al. 2024).

## The Geroscience Perspective on One Health

Geroscience is a multidisciplinary approach to the study of the biology of aging, which seeks to identify the genetic, environmental, dietary, and pharmacological factors that influence the onset of disease, frailty, and disability in late life. Healthy aging has been described as “the ultimate preventative medicine” (Kaeberlein et al. 2015), and therefore it is an aspiration that ought to be prominently incorporated into the One Health concept. The One Health concept invites medical researchers and policymakers to expand their imagination beyond the confines of conceptualizing human disease and health in isolation from extrinsic risk factors (e.g., environmental) and to identify and address the complex interconnections among human, animal, and environmental health.

The concept of the exposome, first introduced by Wild (2005) and expanded on by many others (Vermeulen et al. 2020, Vineis et al. 2020, Ottinger and Geiselman 2023), provides a helpful explanatory framework for making vivid the interdependent connections between environmental hazards and exposures and healthy or unhealthy aging. Wild (2005) introduced the concept of the exposome to help bring some precision to an individual's environmental exposure, to match the methods used for the genome. He contended that, at its most complete, the exposome encompasses life-course environmental exposures from the prenatal period onward, including lifestyle factors. The exposome concept has been expanded to also explicitly acknowledge that social factors tend to shape the exposome, such as historical racism (Nwanaji-Enwerem et al. 2021) and socioeconomic disadvantage (Vineis and Barouki 2022). This life-course exposure explanatory framework is congruent with newly emerging discoveries concerning the epigenetics of the rate of biological aging.

The aberrant gene expressions that can lead to genomic instability are influenced by the lifelong exposure of environmental and lifestyle factors that limit healthspan and lifespan. GPoOH therefore brings to the fore the interdependence of healthy aging and environment. Many decades of research have established a link between increased air pollution and shorter lifespans. Improving the quality of air can improve healthy longevity, as well as benefit animal and environmental health. In their study of 211 county units in the 51 US metropolitan areas with matching data on fine-particulate air pollution for the late 1970s and early 1980s and the late 1990s and early 2000s, Pope and colleagues (2009) found that reductions in air pollution accounted for as much as 15% of the overall increase in life expectancy in the study areas. Long-term exposure to air pollution is also associated with biological aging measures, potentially in a sex-specific manner (Ward-Caviness et al. 2016). In a countrywide US cohort study, air pollution was found to be associated with DNA methylation alterations consistent with higher epigenetic aging among Black but not among non-Hispanic White women (Koenigsberg et al. 2023). Such findings reveal the complex interconnections between environmental exposures and the healthy aging of different groups of a population. And the harm from such environmental exposure extend to nonhuman life. Pollutants can disrupt ecosystem functioning, harming flora and fauna through physiological stress and toxicity, as well as habitat loss (Ogwu et al. 2024).

The concept of green infrastructure (Nieuwenhuijsen 2021) is another useful frame GPoOH can adopt to reveal the interdependence between human and animal and environmental health. Unlike simply the presence of green spaces—such as local parks, trees on streets, and community gardens, all of which can improve human physical and mental health—*green infrastructure* refers to “an interconnected network of greenspace that conserves natural ecosystem values and functions and provides associated benefits to human populations” and “is the ecological framework needed for environmental, social, and economic sustainability” (Benedict and McMahon 2002).

The empirical evidence for the specific health benefits of green infrastructure is still provisional and incomplete. For example, in their analysis of 55 peer-reviewed articles addressing this issue, Venkataramanan and colleagues (2019) found that more familiar types of green investment, such as green spaces and trees, were beneficial to the cardiovascular system, cortisol regulation, and pregnancy health, as well as to mental health and prosocial behaviors. However, in their review, they found that, for newer types of green infrastructure, such as rain gardens, green roofs, and bioswales, there was scant evidence of their impact on health, because few studies have examined the impacts of newer forms of green infrastructure on specific human health outcomes. Furthermore, research on green gentrification reveals the potential for green spaces to exacerbate health disparities—for example, when low-income residents have a lower proportion of tree cover (Landry and Chakraborty 2009). Cole and colleagues (2017) propose a green gentrification and health equity model to help guide policymakers through the complex social and political contexts to ensure equitable and sustainable benefits for all.

## Climate change in an aging world

Depending on the action taken to reduce greenhouse gas emissions, NASA models (NASA 2024) estimate that the global temperature can be expected to rise between 2.5 degrees Celsius (°C) and 4.5°C by the year 2100. By that same year, global fertility is expected to decline below replacement levels, to 1.9 children per woman (UN 2020), and there will be an estimated 3.1 billion people age 60 or over, including nearly 20% of the population in Africa (the world's youngest continent; UN 2021). The future will be both older and warmer (Farrelly 2023c), and this necessitates that more collaborative research be undertaken at the intersection of climate change and aging, what Haq and Gutman (2014) called *climate gerontology*.

Older people are among those most vulnerable to nonoptimal temperatures, including the increased mortality risks of global warming (Hajat et al. 2014, Chen et al. 2024). New research has begun to examine the impact ambient outdoor heat has on accelerating biological aging (Choi and Ailshire 2025). And although estimates of the impact climate change might have on future life expectancy at birth are subject to many uncertainties (and should therefore be taken with some caution), a recent *PLOS Climate* study estimated that an annual average temperature increase of 1°C would reduce global life expectancy at birth by 0.44 years and that such a change disproportionately reduces the life expectancies of women more than the life expectancy of men (Roy 2024).

Urban trees can mitigate both air pollution and urban heat at the neighborhood scale (Yang et al. 2023) and, therefore, are an obvious mitigation strategy prescription of One Health. Urban trees are also vital to the health and survival of the wildlife that live in such environments, such as birds, racoons, deer, and bears, providing shelter, food, places for successful nesting, and resting spots during migration. Research at the intersection of the macrogeophysical environmental level and population health can help reveal the different strategic investments that can be made in improving health for humans, wildlife, and ecosystems.

## The social environment

The social aspects of the environments in which people live also affect healthy or unhealthy longevity. Poor social connections, such as infrequent interactions with others and loneliness, adversely affects mental health and cognition. In a recent study of data from 40,006 participants in 13 longitudinal cohort studies of aging in North America, South America, Europe, Africa, Asia, and Australia, it was found that being in a relationship or married predicted slower global cognitive decline; living with others predicted slower global cognitive, memory, and language decline; and weekly interactions with family and friends and weekly community group engagement predicted slower memory decline (Samtani et al. 2022). Successful aging is influenced by the social environments in which populations age, and these are modifiable factors. During the COVID-19 pandemic, for example, prolonged mitigation measures reduced the social interaction people had with others, increasing the prevalence of loneliness (Baarck et al. 2022).

The One Health concept's emphasis on the interdependence between human and animal health reveals the important role companion animals can play in helping to promote both the physical and mental health of people but especially older people. Owning a pet has been associated with lower levels of social isolation (Kretzler et al. 2022) and may improve the cognition of older adults; some research has shown that pet ownership is associated independently with better cognitive function (Friedmann et al. 2020). Human–animal interaction (including animal-assisted therapy) can confer many health and life satisfaction benefits on older adults, although research on this front is complicated and fraught with challenges (Gee et al. 2017).

## Socioeconomic factors

The socioeconomic environments of modern societies also influence the health disparities and healthspan of a population. Although it has long been documented that higher income is associated with greater longevity (Chetty et al. 2016), more recent research has begun to shed light on how the stressors of socioeconomic disadvantage are implicated in the mechanisms of aging that lead to higher burdens of disease, such as cardiovascular disease, diabetes, cancer, and dementia. Research using a novel DNA methylation measure suggests that children growing up in more disadvantaged families and neighborhoods exhibit a faster rate of biological aging (Raffington et al. 2021). Such empirical findings support the conjecture that cultural features of the environment in which populations live and develop—such as racialized and economic injustice—may contribute to differential rates of biological aging (Krieger et al. 2024). Black women, for example, may be more vulnerable to stress-related disorders and neurodegenerative disease because discrimination accelerates the epigenetics of aging (Elbasheir et al. 2024).

Community-based health promotion interventions for older adults, such as preventative home visits, must be responsive to ethnic and socioeconomic diversity among the target population, and participatory programs that emphasize trust, proximity, and presence have been identified as fundamental factors for improving the acceptability and accessibility of such services (Kristiansen et al. 2021). Intergenerational and community-based group activity programs can help older people’s connection with younger adults and their engagement in social activities, improving their health and psychosocial well-being (Suragarn et al. 2021). And exposure to natural environments is associated with restorative mental health benefits (Pearson and Craig 2014), as well as improvements in cognitive function such as in cognitive-flexibility and attentional-control tasks (Schertz and Berman 2019). However, a greater frequency of everyday discrimination is associated with less satisfaction with experiences in nature (Schinasi and Lawrence 2024), reinforcing the importance redressing persistent socioeconomic inequalities must have in any long-term strategy to improve healthy lifespan.

A One Health perspective on the human and environmental impact of a political economy and culture that create significant economic disparities can help draw greater attention to important public health and ecological considerations often marginalized by a myopic fixation with simply growing gross domestic product, without giving adequate consideration to concerns of distributional fairness and environmental stewardship.

## Lifestyle factors

In addition to external factors such as pollution, exposure to heat, social connections, and socioeconomic disadvantage and inequality, lifestyle factors have a significant impact on healthy or unhealthy longevity. And these factors can be influenced by the environments in which populations live. Drawing on studies employing several experimental models, such as bed rest and reduced step count, Pinto and colleagues (2023) maintained that excessive and prolonged sedentary behaviors can lead to insulin resistance; vascular dysfunction; a shift in substrate use toward carbohydrate oxidation; a shift in muscle fiber from oxidative to glycolytic type; reduced cardiorespiratory fitness; a loss of muscle mass, strength, and bone mass; and increased total body fat mass, visceral fat depot, blood lipid concentrations, and inflammation. Large cohorts of people are employed in sedentary occupations, and the time spent sitting and being physically inactive at work has increased in recent decades (Shrestha et al. 2018).

Furthermore, the design of urban settings that prioritize heavy motorized vehicle use for travel (e.g., to work, schools) can limit a population's opportunity to walk or cycle, with determinantal impacts on both human and environmental health (e.g., increasing greenhouse gas emissions from automobiles). The World Health Organization's guide to age-friendly cities proposes a bottom-up participatory approach to optimizing the opportunities for health, participation, and security to enhance quality of life as people age (WHO 2007). The guide is focused on eight topic areas: transportation, housing, social participation, respect and social inclusion, civic participation and employment, communication and information, community support and health services, and outdoor spaces and buildings. The guide notes the importance of measures such as making pavement age-friendly by ensuring a smooth, level, and nonslip surface; sufficient width to accommodate wheelchairs; and dropped curbs that taper off to be level with the road. I also recommends outdoor seating areas where pedestrians have the opportunity to rest, as well as adequate and accessible public toilets. A One Health approach to healthy aging could complement WHO's emphasis on age-friendly cities, encouraging the pursuit of cost-effective modulations to the physical designs of modern cities in ways that promote human, animal, and environmental health.

A similar point can be made with respect to diet and nutrition (Kritchevsky 2016). The amount of calories a person typically consumes, as well as the nutritional content of those calories (e.g., sodium, iron, calcium), can affect their health in different ways, increasing or decreasing their risks of hypertension, heart disease, cancer, dementia, and so on. Some traditional and contemporary dietary patterns, such as the Mediterranean diet and the DASH Eating Plan (for *dietary approaches to stop hypertension*) have been associated with lower mortality and healthy longevity (Hu 2024). Creating healthier food environments, such as replacing the abundance of highly saturated foods with more options for plant foods and culinary herbs and spices, would be beneficial to human, animal, and environmental health. The same would be true if human populations reduced the amount of food typically consumed, especially ultraprocessed foods that place pressure on the diversity of plant species available for human consumption (Leite et al. 2022). The UN (2022; https://news.un.org/en/story/2022/12/1131637) contends that Europe and North America consumed the most calories in the year 2021, at 3540 per day, whereas African nations consumed the least, at 2600 calories per day. The One Health concept helps reveal the interconnectedness between the unhealthy consumption habits of the world's richest countries and the environmental impact such habits can have on people living in other parts of the world, where the climate change impacts may be more harmful because of geographical vulnerabilities.

The interventions emphasized above pertain to, for the most part, what we would now call, in Herter's terminology, elements of *humanitarian medicine*. These are the direct and obvious measures of preventative medicine that can improve human health by reducing the life course exposure to the environmental stressors that can accelerate the rate of biological aging. A few decades ago, many of today's basic public health prescriptions, such as smoking cessation and fostering social connections, were still scientific speculation in need of more experimental data before moving from the realm of curiosity-oriented science and into humanitarian medicine. This means our understanding of which public health interventions are considered direct and obvious changes over time, as our knowledge and understanding of biology, pathology, and life course exposures improve. Today, we often take for granted sanitation, antibiotics, and vaccinations, but these measures were the experimental science of yesterday.

Environmental exposures such as pollution and solar ultraviolet radiation, diet, and exercise can all affect, in both positive and negative ways, the healthy aging of the world's populations. Gene–environment interactions may play a substantial role in accelerating the rate of biological aging and its associated risks of adverse aging-related outcomes (Malecki et al. 2022). For most of these interventions, such as air pollution reduction, enhancing the green infrastructure, improving the quality of dietary options, and the opportunities for exercise and connecting with people and nature, the health dividend to human populations would also likely yield significant improvements to animal and environmental health.

But the environmental impact of the healthy life extension that may be achievable via potential pharmacological interventions in aging is more challenging to both estimate and communicate (e.g., to the general public and policymakers). There is still uncertainty not only about their potential safety and efficacy but also about the likely magnitude of their impact on human healthspan and lifespan. If experimental gerontology is perceived to be a public health strategy that could significantly increase the lifespan of human populations, regardless of the potential impact on animal and environmental health, it may be construed as something that conflicts with, rather than supports, the One Health concept. We now address these issues in the following two sections of this Forum article.

## Experimental gerontology and lifespan extension

Biomedical research to treat or prevent a specific disease (e.g., cancer, Alzheimer's, or COVID-19) is intuitively perceived by the general public and policymakers as a morally laudable scientific aspiration. Such interventions are framed and communicated as “saving lives”. However, the societal benefits of drugs that slow the rate of biological aging are not intuitive. No one officially dies from aging (Hayflick 2003, Hayflick 2020), and therefore, research on the biology of aging may be conceived of as yielding translational science that merely extends life. That is an end that, at best, is ambiguous, because it is not clear whether it is an extension of *healthy* life or the period of time older populations survive by managing multimorbidity, frailty, and disease. And, at worst, the stated policy aspiration of extending life can prime sentiments of what Richard Miller (2002) calls *gerontologiphobia*—“the irrational public predisposition to regard research on specific late-life diseases as marvelous but to regard research on aging and thus all late-life diseases together as a public menace bound to produce a world filled with nonproductive, chronically disabled, unhappy senior citizens consuming more resources than they produce.” Gee and Gutman (2000) challenge the common presumptions of apocalyptic demography in their book *The Overselling of Population Aging: Apocalyptic Demography, Intergenerational Challenges,*  *and Social Policy*.

Returning to Herter's distinction between humanitarian medicine- which encompasses interventions that already have an established empirical track record of helping to abate disease, suffering, and mortality (such as traditional preventative medicine)- and experimental science- the still unproven but potentially novel ways of promoting health- we can now consider the potential impact on human–animal–environmental health of potential pharmacological interventions from translational gerontology.

This field of scientific research is largely unknown to many medical researchers outside the fields of geriatric medicine and gerontology. But it is an area of research with a long history of experimental and biological science behind it. Insights from comparative biology reveal that there is significant variation in the lifespan of animals. The roundworm *Caenorhabditis elegans*, the first animal genome sequenced in 1998 (*C. elegans* Sequencing Consortium 1998), has a mean lifespan of approximately 18–20 days when cultured at 20°C (Zhang et al. 2020). By contrast, the naked mole rat (*Heterocephalus glaber*) lives at least 17 years in the wild and almost twice that in captivity and avoids many of the age-related changes (e.g., cancer) that are prevalent in other species (Buffenstein 2008, Austad 2009). Larger breeds of dogs have a shorter lifespan—by about 5 years on average and up to 8 years—than smaller dogs do (Kraus et al. 2023). And the hydra, which possesses a robust regenerative capacity that enables it to constantly renew its tissues, is considered biologically immortal (Martínez 1998, Johnson et al. 2019).

Observations of the diversity in animal lifespans lead many biologists to theorize about the evolutionary explanation for senescence. Building on the insights of researchers such as J. B. S. Haldane, Peter Medawar, George Williams, and William Hamilton, two main theories of the evolution of aging were advanced in the twentieth century: the mutation accumulation theory of aging (suggested by Medawar 1952) and the antagonistic pleiotropy theory of aging (suggested by Williams 1957). The dominant version of the latter explanation is known as the *disposable soma theory* (Kirkwood 1977, Kirkwood and Holliday 1979); the theory posits that the winning evolutionary strategy for sexually reproducing species, species that have had to find ways to survive the extrinsic risks of the hostile environments of life, is to prioritize health during the *essential span*—that is, the time required for successful reproduction and continuation of generations (Rattan 2000).

Understanding why biological aging occurs, from an evolutionary perspective, is itself a significant advancement in biological knowledge. But the twentieth century also yielded significant empirical findings regarding the prospect of intentionally modulating the rate of biological aging. And knowledge about, and experiments with, the healthy aging of animal models has been critical to translational gerontology (Ottinger and Holmes 2024). The foundational research came from experiments in caloric (or dietary) restriction in rodents, although the initial lines of research studied the influence of dietary intake on cancer and the effect of malnutrition early in life on growth, reproduction, and adult health (Anson et al. 2005). Rous (1914) found that a reduction of food intake of a special diet and sometimes simple underfeeding reduced the incidence of cancer in rodents. Around the same time, Osborne and colleagues (1917) examined the impact retardation of growth had on the duration of the life of rats. And McCay and colleagues (1935) published their seminal study, which provided data to show, for the first time, that a restriction of calories without causing malnutrition prolongs mean and maximal lifespan in rats. And research conducted on caloric restriction in mice during the 1970s and 1980s demonstrated conclusively that caloric restriction has a major impact on aging by preventing or delaying the incidence of most age-related diseases and pathologies in rodents (Richardson 2021). Research in biomedical gerontology is significant not because it extends the duration of life but because it increases healthspan.

The empirical evidence on both the potential feasibility and the potential safety of caloric restriction in humans, or some other fasting regimes designed to modulate biological aging, is something researchers have only recently begun to explore. However, an intervention such as caloric restriction would have to be long term, making it too burdensome of an intervention to expect people to comply with. But the scientific value of animal laboratory experiments with caloric restriction lays not with their potential to provide data or insights to derive stringent dietary restrictions on the human diet but, rather, to aid with the development of drugs (*gerotherapeutics*) that mimic the effects of dietary restriction to delay the onset of disease, frailty, and disability in late life (de Cabo and Mattson 2019).

In the 1980s, the era of genetic manipulations in aging in invertebrates and mice demonstrated that genetic mutations lead to increased lifespan (Richardson 2021). Like caloric restriction, it is not going to be feasible—at least for the foreseeable future (if ever)—to genetically engineer humans to increase healthy longevity, because of concerns of both safety and cost. But the most significant development, which transformed translational gerontology from the realm of mere speculation to the new frontier of public health (Kirkland et al. 2023), occurred in the early 2000s. And this was the discovery of drugs that mimic the lifespan benefits associated with caloric restriction, referred to as *calorie restriction mimetics* (Madeo et al. 2019, NIA 2020).

In 2004 The National Institute on Aging's Interventions Testing Program (ITP), a peer-reviewed program designed to identify agents that extend lifespan and healthspan in mice, was created (Nadon et al. 2017). The advent of the era of pharmacological and pharmaceutical interventions in aging was ushered in when molecules were discovered that could alter the rate of biological aging. And over the next 20 years, a number of compounds were discovered that significantly increase the median lifespan of mice, which demonstrated that lifespan could be significantly modulated by the consumption of a drug. This meant that there could be real medical utility derived from the experimental science of biomedical gerontology.

The first two drugs that were discovered to increase lifespan in mice were metformin (Anisimov et al. 2008) and rapamycin (Harrison et al. 2009). Both drugs were already approved by the US Food and Drug Administration as therapeutics for humans, which means they are off patent and have an extensive track record for safety and so could potentially be repurposed to target biological aging (Snell et al. 2016). Metformin has been used as a pharmacological intervention to control type 2 diabetes for decades, and rapamycin is a drug used to help prevent the rejection of transplanted organs for patients undergoing organ transplant.

Progressing translational gerontology from discovering compounds that extend healthspan and lifespan in laboratory animals to determining whether they are safe and effective in humans is the next major challenge for geroscience. This innovative medical research exemplifies the experimental science that Herter hailed back in the early twentieth century. But how does this arm of experimental science fit into the One Health concept? Improving human health through healthy environments (e.g., green spaces) and lifestyle (e.g., exercise) encapsulates the ethos of planetary health stewardship of One Health. But the prospect of drugs that could significantly increase lifespan, even if it is a healthy lifespan, raises concerns about the ecological impacts of extending the duration of life for the more than 2 billion humans that will be over age 60 by the year 2050. Does the experimental arm of geroscience represented by the ITP represent a kind of moral hazard to the One Health concept?

## Would gerothereaputics constitute a moral hazard to One Health?

In economics, a *moral hazard* is a risk that someone may be more inclined to take because they do not expect to bear the full costs of their actions, because they have insurance to protect them from such harms. Adam Smith (1776) noted such a hazard in *The Wealth of Nations*, claiming that the directors of companies such as the Bank of England, because they manage other people's money rather than their own, cannot be expected to watch over such funds with the same anxious vigilance that partners in a private company managing their own funds would have. If gerotherapeutics offer the potential to extend the healthy lifespan of the world's aging populations, will this undermine support for the nonpharmacological interventions that can promote animal and environmental health? In other words, with access to gerotherapeutics to aid them in achieving healthy longevity, is there not a risk that people will continue to overconsume and live sedentary lifestyles, secure in the knowledge that they have access to drugs that will prolong healthy longevity without worrying about the environmental interventions or lifestyle behaviors that could improve planetary health?

The recent development of antiobesity drugs (Wilding et al. 2021), such as Ozempic, has already amplified this type of worry and provides the basis for a reasoned response to the objection that gerotherapeutics would constitute a moral hazard to One Health. Antiobesity drugs (just like potential gerotherapeutics) are not a magic pill for weight loss (or healthy longevity). At best, antiobesity medication can supplement but not replace the nonpharmacological interventions of traditional preventative medicine, such as a healthy diet and exercise. And humans will still require clear air and water; access to green spaces; connections with other people, animals, and nature, and so on, for optimal physical and mental health, with or without access to gerotherapeutics.

But GPoOH can go further than just this type of minimal response to the moral hazard concern. Gerotherapuetics may also be beneficial to animal and environmental health, if deployed intentionally for such purposes (Farrelly 2024). Indeed, this a central prescription of GPoOH, because it is not only concerned with improving human health. GPoOH recognizes that reducing the nearly 10 year gap between human healthy life expectancy and life expectancy could reap both direct and indirect benefits to animal and environmental health.

There are good reasons for believing that the development of aging drugs could directly improve the health of both companion and agricultural animals. Companion animals such as dogs and cats biologically age and therefore suffer the health maladies of senescence. And a significant portion of the veterinary medicine for companion animals is devoted to managing the health maladies that arise because of the disparity between lifespan and healthy lifespan. Gerotherapuetics may be a health intervention for companion pets, as well as humans.

The Dog Aging Project (https://dogagingproject.org/), a long-term longitudinal study involving tens of thousands of companion dogs, seeks to identify the genetic, environmental, and lifestyle factors associated with a healthy lifespan (Creevy et al. 2022). And one study of that project is the TRIAD (Test of Rapamycin in Aging Dogs), which involves assessing whether the consumption of the molecule rapamycin, which increases the lifespan and healthspan of mice, has a similar effect in dogs. This study is a nice illustration of the potential experimental gerontology has to directly benefit both animal and human health.

The potential application of gerotherapeutics could also be extended to agricultural animals, such as dairy cattle (Farrelly 2024). The average productive lifespan of dairy cattle is only 3–4 years in countries with high-producing dairy cows (De Vries and Marcondes 2020), but their estimated maximum lifespan is about 20 years (Hoffman and Valencak 2020). After their ability to produce milk diminishes, dairy cattle are slaughtered for meat. The disparity between their lifespan and productive lifespan means that many more dairy cattle must be bred, fed, and raised to produce the same amount of dairy products that could be yielded by a smaller number of dairy cattle with a longer productive lifespan. Translational gerontology might help reduce the amount of greenhouse gas emissions from dairy cattle by extending the period of their health and productivity. This would be so if less cattle feed (and therefore greenhouse gas emissions) would be needed to yield comparable levels of milk to that produced by a higher number of shorter-lived cattle. Dairy cattle do not produce milk before they give birth to their first calf, around age 2. Rearing higher numbers of cattle for dairy production is a more feed-intense process than extending the productive lifespan of adult cattle that can produce the same quantity of milk. As such, the smaller the disparity between the productive lifespan and lifespan of dairy cattle, the less determinantal the impact on animal and environmental health.

Perhaps the most important point to note about this still experimental element of GPoOH is that a reduction of the disparity between human lifespan and healthspan would significantly reduce the greenhouse gas emissions from the global healthcare systems of an aging world. For the OECD (Organisation for Economic Co-operation and Development) countries, China, and India, health care on average accounts for 5% of the national carbon footprint, comparable in importance to the food sector (Pichler et al. 2019). If the rate of biological aging is not modulated to increase healthspan, then medical innovations that increase lifespan by extending the duration of life by managing multimorbidity will increase the environmental impact of the world's healthcare systems. In other words, as the disparity between healthy life expectancy and life expectancy grows, this will have an adverse impact on planetary health more generally.

The economic benefits of slowing aging would also be significant (Goldman et al. 2013, Olshansky 2016), helping the world's economies be better positioned to invest in renewable energies and other mitigation strategies, as well adapt to climate change. Healthier older populations mean savings on healthcare expenditures, a more productive workforce, and human capital development (Rowe 2015). GPoOH prioritizes the goal of redressing the disparity between life expectancy and healthy life expectancy by pursuing interventions and innovations that increase healthspan in environmentally sustainable ways.

## Conclusion

Herter emphasized the importance of understanding science as a plastic moral and intellectual enterprise. In this Forum article, the case has been made for championing the Geroscience Perspective on One Health, which is predicated on a malleable understanding of the One Health concept. Three elements of the plasticity of GPoOH are worth emphasizing in conclusion. First, One Health can encompass a concern for abating the chronic, and not just infectious, diseases (e.g., heart disease, cancer) more prevalent in late life. By doing so the One Health concept draws attention to the diverse preventative measures that can improve healthy aging, as well as animal and environment health.

Second, by adopting the explanatory framework of the exposome, as well as the latest scientific findings on drug interventions into the aging of laboratory animals, GPoOH highlights the importance of both nonpharmacological and potential pharmacological interventions that can slow aging and improve healthspan. And, finally, GPoOH is also plastic in that it adopts a broadly hopeful but still realistic communicative frame about healthy aging and animal and environmental health. This is a departure from communicative strategies designed to amplify climate anxiety (Ugargol and Poladi 2024), intergenerational conflict, and emotions such as anger and guilt (Lu and Schuldt 2015). GPoOH does not catastrophize global demographic changes, such as population aging. But it does highlight both the opportunities and challenges presented by population aging. In particular, it draws attention to the reality that a growing disparity between healthy life expectancy and life expectancy is detrimental for human, animal, and environmental health.

Admittedly, the account of GPoOH advanced in the present article is only a starting point, and there are many other issues that it needs to address. For example, if extending healthspan via drug intervention is accompanied by an increase in lifespan, then issues such as housing and feeding growing older populations must take seriously the implications this would have on animal and environmental health. This does not mean we should forfeit the health innovations of geroscience, but the pursuit of healthy longevity must be done responsibly, with both foresight and a moral concern for the impact on other animals and the environment.

Furthermore, the implications of geroscience for companion and agricultural animals addressed in the present article have been very limited, leaving aside the much broader issue of the implications it may have for wildlife, both animal and vegetal. GPoOH emphasizes how research on extending the healthy lifespan of laboratory animals can help with the innovation of drugs to increase human healthspan. But how can this science also help improve wildlife? A few examples have been advanced in this article, such as research at the intersection of the macrogeophysical environmental level and population health, as well as alterations to the human diet and consumption habits. Future collaborative research between climate science and geroscience, especially when collaboratively pursued through the lens of One Health, would help to shed light on further points of overlap between these two areas of study.

Science communication is political communication (Scheufele 2014), and the media's negativity bias exerts pressure on scientists to communicate the societal urgency of accelerating translational science in ways that often risk perpetuating ageism. By eschewing narratives of intergenerational conflict and the sentiments of gerontologiphobia (Miller 2002), GPoOH highlights the humanitarian medicine and experimental science that may improve human, animal, and environmental health. As such, it has important contributions to make to the One Health concept and to science communication.

## Notes

Colin Farrelly (farrelly@queensu.ca) is affiliated with the Department of Political Studies at Queen's University in Kingston, Ontario, Canada.
